# Glyphosate (Ab)sorption by Shoots and Rhizomes of Native versus Hybrid Cattail (*Typha*)

**DOI:** 10.1007/s00128-017-2167-6

**Published:** 2017-09-14

**Authors:** Tianye Zheng, Nora B. Sutton, Pim de Jager, Richard Grosshans, Sirajum Munira, Annemieke Farenhorst

**Affiliations:** 1 Department of Electrical Engineering, The Hong Kong Polytechnic University, Kowloon, Hong Kong; 2 Sub-department of Environmental Technology, Wageningen University and Research Centre, Bornse Weilanden 9, Wageningen, The Netherlands; 3 International Institute for Sustainable Development (IISD), 111 Lombard Avenue, Suite 325, Winnipeg, MB Canada; 4 Department of Soil Science, University of Manitoba, 380 Ellis Building, Winnipeg, MB Canada

**Keywords:** Phytoremediation, Wetland pollution, Native cattail, Hybrid cattail, Glyphosate, Sorption

## Abstract

Wetlands in the Prairie Pothole Region of North America are integrated with farmland and contain mixtures of herbicide contaminants. Passive nonfacilitated diffusion is how most herbicides can move across plant membranes, making this perhaps an important process by which herbicide contaminants are absorbed by wetland vegetation. Prairie wetlands are dominated by native cattail (*Typha latifolia*) and hybrid cattail (*Typha x glauca*). The objective of this batch equilibrium study was to compare glyphosate absorption by the shoots and rhizomes of native versus hybrid cattails. Although it has been previously reported for some pesticides that passive diffusion is greater for rhizome than shoot components, this is the first study to demonstrate that the absorption capacity of rhizomes is species dependent, with the glyphosate absorption being significantly greater for rhizomes than shoots in case of native cattails, but with no significant differences in glyphosate absorption between rhizomes and shoots in case of hybrid cattails. Most importantly, glyphosate absorption by native rhizomes far exceeded that of the absorption occurring for hybrid rhizomes, native shoots and hybrid shoots. Glyphosate has long been used to manage invasive hybrid cattails in wetlands in North America, but hybrid cattail expansions continue to occur. Since our results showed limited glyphosate absorption by hybrid shoots and rhizomes, this lack of sorption may partially explain the poorer ability of glyphosate to control hybrid cattails in wetlands.

Pesticide contamination in surface water has been frequently reported (Hiller et al. 2008; Messing et al. 2013, 2014b; Richards and Baker 1993). Glyphosate [*N* (phosphonomethyl) glycine] and MCPA (2-methyl-4-chlorophenoxyacetic acid) are among the most widely applied herbicides in North American agriculture, and are frequently detected in surface waters, particularly in wetlands that are integrated with farmland in the Prairie Pothole Region of North America (Benbrook 2016; Farenhorst et al. 2015a, b; Messing et al. 2014a; Schrübbers et al. 2016). Prairie wetlands are contaminated with a range of pesticides that can enter wetlands because of spray drift, atmospheric wet and dry deposition, gas exchanges at the air–water interface, surface runoff and groundwater recharge (Messing et al. 2011; Waite et al. 1992, 1995). The concentrations of individual pesticides detected in Prairie wetlands are typically in the parts per billion range with a reported maximum detection of 9 μg/L for MCPA (Donald et al. 1999). In surface waters, pesticides can be present in the parts per million range with reported maximum detections of 1.48 mg/L for glyphosate (Mayakaduwa et al. 2016) and of 0.29 mg/L for other pesticides including MCPA (Ignatowicz 2009).

Conventional physical and chemical technologies for removing contaminants from surface water aim at removal before utilization, for example, flocculation, membrane filtration (Shkinev 2001). Some technologies can be effective (e.g. reverse osmosis, chemical precipitation), but with high investment and operational costs (Olette et al. 2008; Pearson 2001). Consequently, these technologies are typically applied for producing potable water, not for mitigating surface water quality. Phytoremediation within the context of (constructed) wetlands is an alternative technique that can be applied to mitigate the concentrations of a range of contaminants in water including pesticides, nutrients, heavy metals, and pharmaceuticals (Dosnon-Olette et al. 2009). These more nature-based technologies have the advantages of being low cost, easy to operate and having a low environmental impact. In previous studies, the capacity of some aquatic plants (e.g. duckweed) and algae species to absorb organic contaminants has been investigated (Dosnon-Olette et al. 2010a, b). Less information (Moore et al. 2013) is available on cattail (*Typha*) species, which is notable, considering that these plants often dominate wetlands. Prairie wetlands in North America are dominated by a mix of native cattail (*Typha latifolia*), the European introduced cattail (*Typha x angustifolia*) and hybrid cattail (*Typha x glauca*) from the two parent species. The native (*Typha latifolia*) and the hybrid species (*Typha x glauca*) were chosen to be tested in this study.

Since the primary pathway for organic contaminants to enter plants is via passive absorption (Li et al. 2005; Ma and Wang 2009), it is crucial to understand the sorption characteristics of different species of cattails. The objective of this batch equilibrium study was to investigate the absorption of glyphosate and MCPA by the shoots and rhizomes of native versus hybrid cattails. These results can provide important information on the removal of pesticides by different cattail tissues as well as giving an indication of the susceptibility of cattail species towards pesticides.

## Materials and Methods

Herbicides purchased were [phosphonomethyl-14C] glyphosate (99% radiochemical purity; specific activity 50 mCi/mmol) and [2-methyl-4-chlorophenoxyacetic acid ^14^C] MCPA (98% radiochemical purity; specific activity 55 mCi/mmol) from American Radiolabeled Chemicals Inc., USA, and analytical grade glyphosate (99.9% purity) and MCPA (99% purity) from Sigma-Aldrich Co., St. Louis, MO, USA. Glyphosate and MCPA properties are given in Table 1. The native cattail species was collected from a wetland located in the Experimental Lakes Area (ELA) of the Province of Ontario, Canada. The hybrid cattail species was collected from the constructed wetland named Pelly’s Lake located in the Province of Manitoba, Canada. The cattail plant samples were separated into native shoots, native rhizomes, hybrid shoots and hybrid rhizomes and then oven-dried (65 °C) for 72 h and pulverized using a grinder.

**Table 1:** Physical–chemical properties of glyphosate (Franz et al. 1997) and MCPA (Hiller et al. 2008)

Herbicides	Molecular weight (g/mol)	Solubility in water at 25 °C (mg/L)	Octanol–water partition coefficient (log K_ow_)	Chemical structure
Glyphosate	169.1	12,000	−3.40	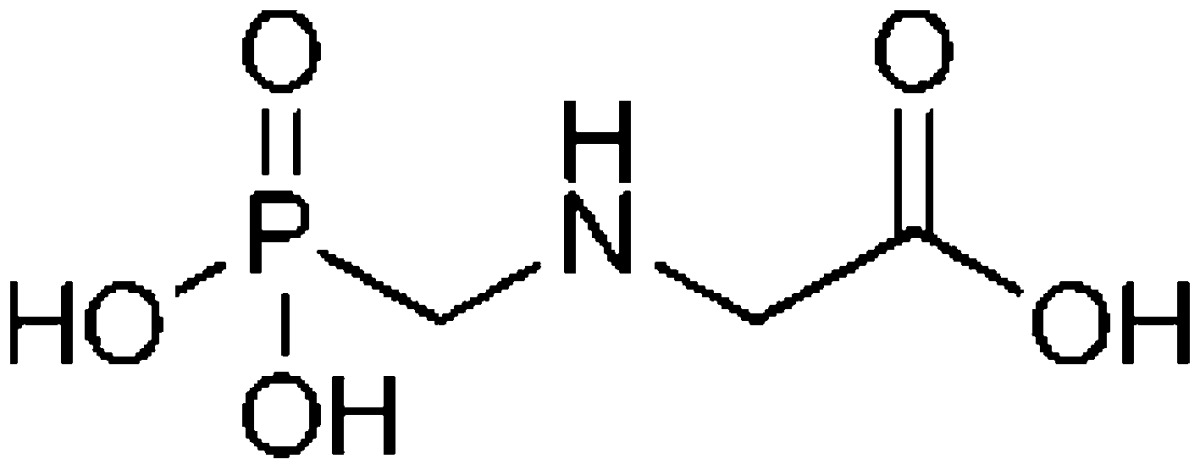
MCPA	200.6	273.9	−0.71	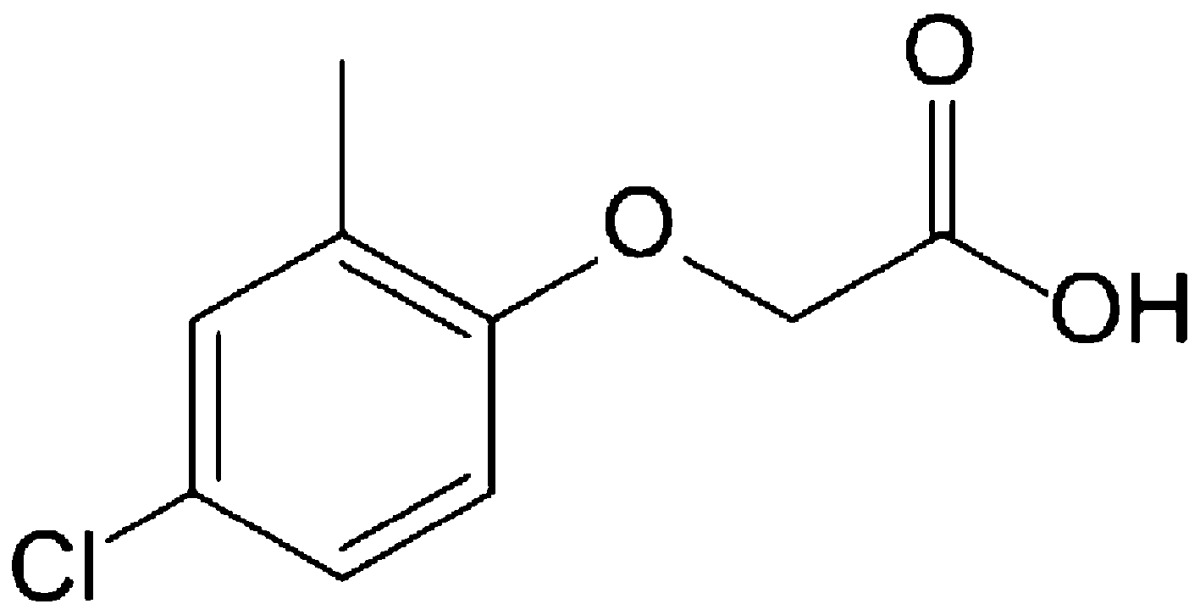

Glyphosate stock solutions consisted of 10 μg/L or 1 mg/L analytical-grade glyphosate and 6.67 × 10^4^ Bq/L ^14^C-labelled glyphosate. MCPA stock solutions consisted of 10 μg/L or 1 mg/L analytical-grade MCPA and 1.67 × 10^4^ Bq/L ^14^C-labelled MCPA. We included MCPA in this study so that we could compare the sorption of glyphosate against that of another herbicide that is frequently detected in Prairie wetlands. Solutions (15 mL) were added to 0.2 g plant samples in 50 mL Teflon tube in triplicates, and a previous batch equilibrium study (Trapp and Miglioranza 2001) has used a similar plant to solution ratio. Slurries were rotated at 5 °C in the dark for 0.5, 1, 2, 4, 8, 24, and 48 h to determine the absorption of glyphosate over time.

For MCPA, only the 48 h time step was used since the absorption was too weak to be analyzed over time. At each time, tubes were centrifuged at 10,000 rpm for 10 min and subsamples (1 mL) of supernatant (duplicates) were added to scintillation vials (7 mL) containing 5 mL 30% Scintisafe scintillation cocktail (Fisher Scientific, Fair Lawn, NJ). Vials were lightly shaken before radioactivity was measured. The radioactivity in the initial solution and centrifuged supernatant were measured by Liquid Scintillation Counting (LSC) with automated quench correction (#H method) (LS 6500 Beckman Instruments, Fullerton, CA) and a maximum counting time of 10 min.

The plant-solution partition coefficients K_pl_ (L/kg) of glyphosate or MCPA was calculated by:

$${K_{pl}}=\frac{{\left( {{C_i} \times V - {C_e} \times V} \right)/{W_{pl}}}}{{{C_e}}}$$

where C_i_ is the concentration of radioactivity in the initial solution; C_e_ is the concentration of radioactivity in the centrifuged supernatant; V is the volume of solution; W_pl_ is the oven-dry weight of plant sample.

Statistical analysis was conducted by using SAS software version 9.3 for Windows (SAS Institute Inc. 2002–2010). Repeated measure analysis and multiple means comparison (Tukey’s) tests were utilized in PROC GLIMMIX to determine the effect of shaking time and plant treatment on K_pl_ values. Two-way ANOVA in PROC GLIMMIX was used to quantify the effect of concentrations (10 μg/L, 1 mg/L) and plant treatments (native shoots, native rhizomes, hybrid shoots, hybrid rhizomes) on K_pl_ values at 48 h.

## Results and Discussion

**Fig. 1:**
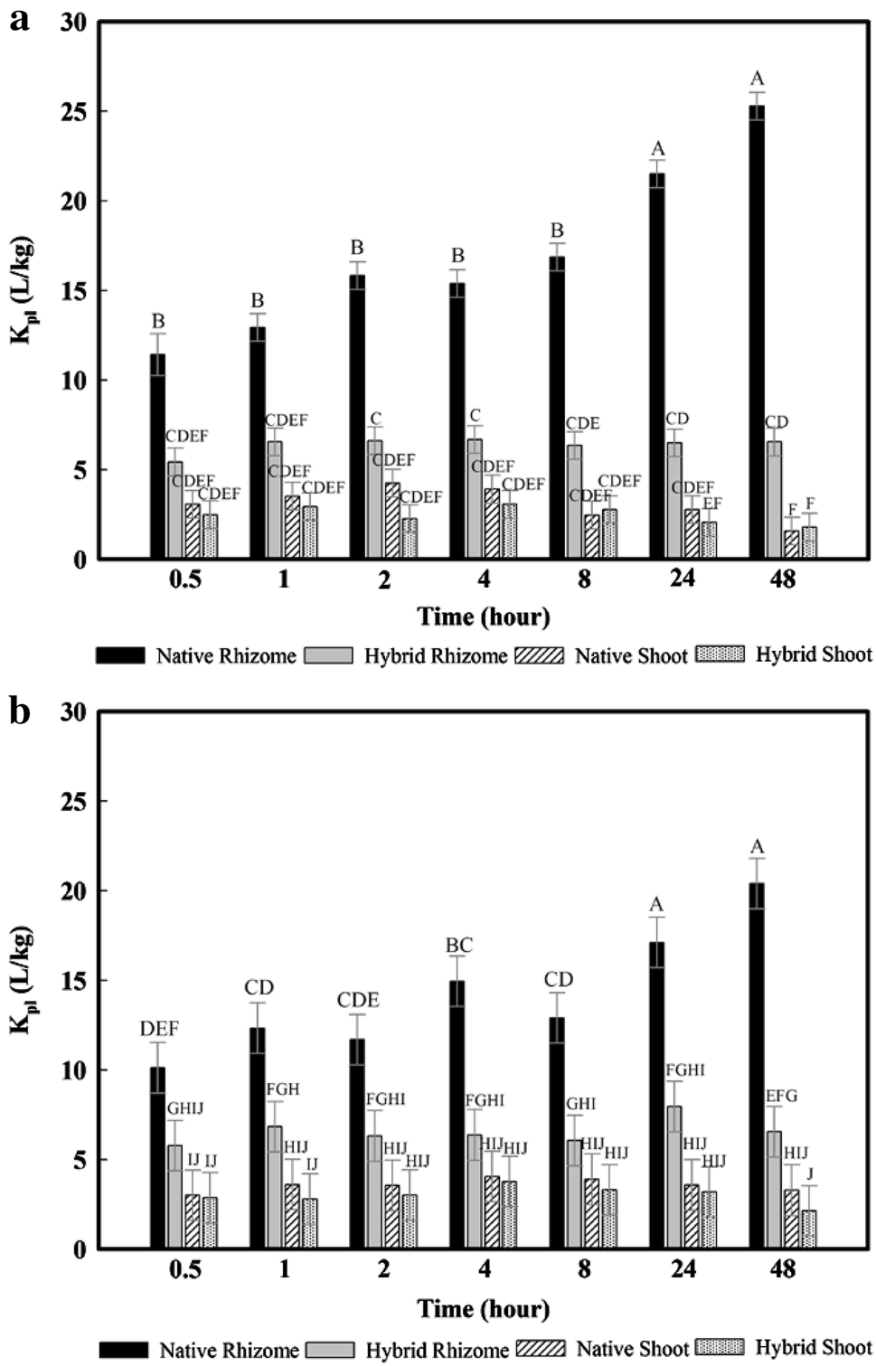
Time dependent sorption study of the plant-solution partition coefficient (K_pl_) of glyphosate at the initial concentration of **a** 10 μg/L and **b** 1 mg/L; the K_pl_ values were calculated by taking the average of the triplicate and the *error bars* indicated the standard deviation; the *letters* obtained from statistical analysis indicate the significance between the K_pl_ values

Regardless of whether the initial solution contained 10 μg/L or 1 mg/L glyphosate, native rhizomes always demonstrated significantly greater glyphosate K_pl_ values than hybrid rhizomes, and than native or hybrid shoots (Fig. 1). In fact, at 0.5 h, the glyphosate K_pl_ values measured for native rhizomes were significantly larger than the glyphosate K_pl_ values measured at any time step for hybrid rhizomes, and for native or hybrid shoots, including at 48 h. For native rhizomes, glyphosate K_pl_ values before 24 h were significantly smaller than after 24 h, suggesting that glyphosate continued to partition into the native rhizomes over time. However, glyphosate sorption approached equilibrium between 24 and 48 h for the native rhizomes because there were no significant differences in glyphosate K_pl_ values between these two time steps. In contrast, regardless of whether the initial solution contained 10 μg/L or 1 mg/L glyphosate, glyphosate absorption reached its maximum within 0.5 h for hybrid rhizomes, as well as for native and hybrid shoots. The reason was that shaking time had no significant impact on glyphosate K_pl_ values for each of these plant treatments (Fig. 1). Also, for most time steps, there was no significant difference in glyphosate K_pl_ values between hybrid rhizomes and native or hybrid shoots.

In a previous batch equilibrium study (Li et al. 2005) that added organochlorines in solution to wheat and ryegrass seedlings, it reported that the passive nonfacilitated diffusion of pesticides was greater for rhizomes than shoots. More importantly, our results indicated that the absorption capacity of rhizomes can be species dependent, with the glyphosate absorption being significantly greater for rhizomes than shoots in case of native cattails, but with no significant differences in glyphosate absorption between rhizomes and shoots in case of hybrid cattails. As shown in our experiment, the time to equilibrium was also dependent on the type of plant materials studied and was rapid (0.5 h) for hybrid rhizomes, and for native and hybrid shoots but relatively slower (24–48 h) for native rhizomes. Equilibrium time is affected by the selection of sorbents and contaminants but is typically set at 24 h in most batch equilibrium studies, particularly in studies of pesticides sorption by soils (Kumari et al. 2016; Munira et al. 2016; Wauchope et al. 2002). In previous batch equilibrium studies, Mayakaduwa et al. estimated an equilibrium time of 4 h when glyphosate in solution was applied to woody biochar (Mayakaduwa et al. 2016). Ma and Wang et al. reported that the sorption of trichloroethylene by cattail roots required 4 days to reach equilibrium (Ma and Wang 2009).

For most herbicides, passive diffusion is an important process by which herbicides can move into roots and then translocate to action sites to adversely affect the plant. Our results indicated that the passive diffusion of glyphosate by rhizomes is less for hybrid than native cattail species, including at relatively large glyphosate concentrations in water. This suggests that hybrid cattails could be less sensitive to glyphosate concentrations in water than native cattails and perhaps explain recent observations in field settings (Linz and Homan 2011). For example, the U.S. Department of Agriculture’s Wildlife Services has applied glyphosate to manage invasive hybrid cattails since last century. Nonetheless, hybrid cattail expansion has remained a critical challenge in North America because hybrid cattails demonstrate resistance towards glyphosate treatment (Linz and Homan 2011). Our experimental results, showing limited glyphosate absorption by hybrid shoots and rhizomes, may partially explain the resistance of hybrid cattails towards glyphosate treatment and the displacement of the native by the hybrid cattail, as sorption is an essential first step in treatment.

**Fig. 2:**
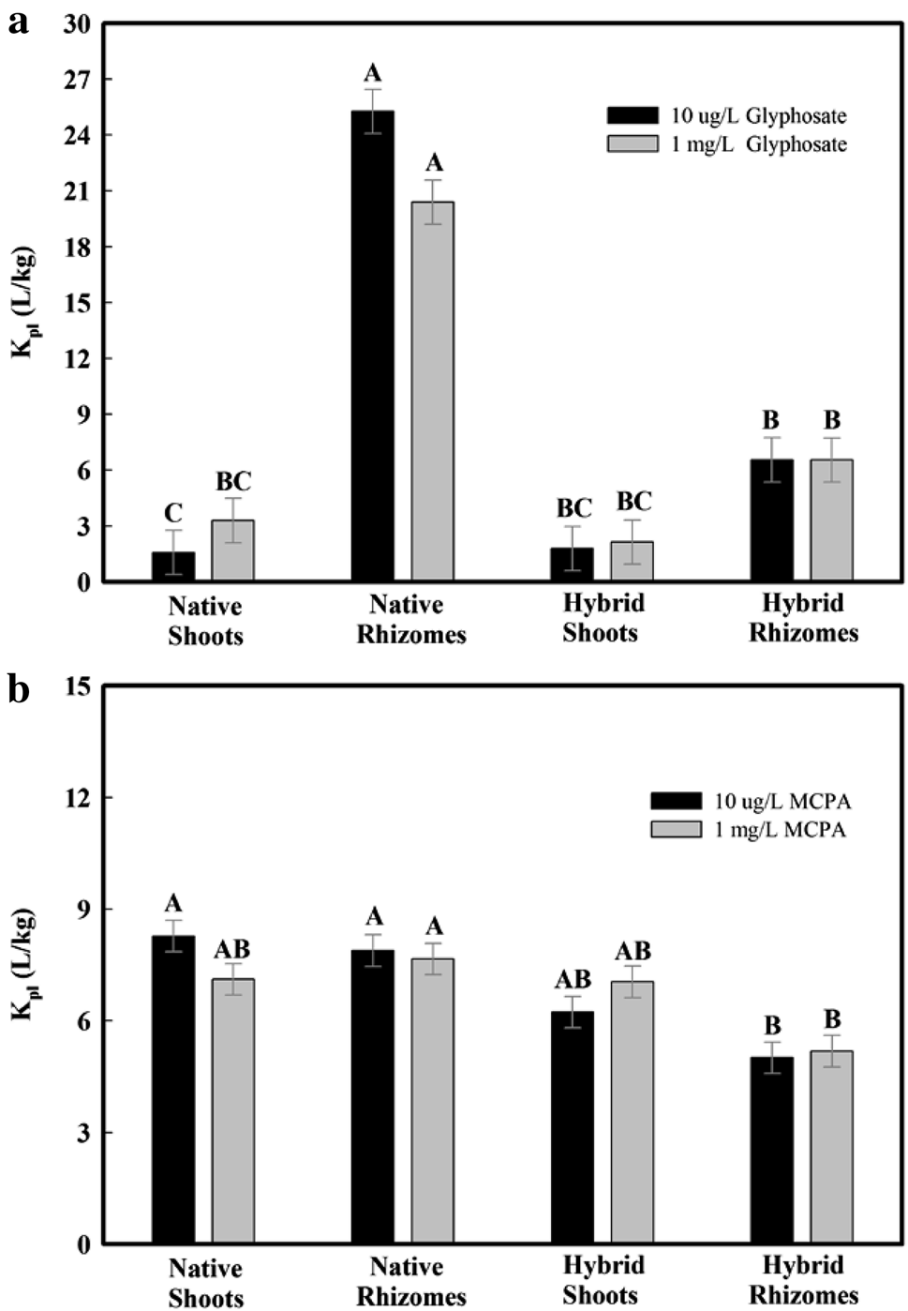
Concentration dependent sorption study of **a** the plant-solution partition coefficient (K_pl_) of glyphosate and **b** the plant-solution partition coefficient (K_pl_) of MCPA after 48 h; the K_pl_ values were calculated by taking the average of the triplicate and the *error bars* indicated the standard deviation; the *letters* obtained from statistical analysis indicate the significance between the K_pl_ values

Sorption data for glyphosate and MCPA obtained at 48 h were further analyzed to help understand the different behaviors of the four cattail treatments at the two chosen concentrations (Fig. 2). Statistical analysis demonstrated that the effect of cattail treatments on K_pl_ values was significant for both glyphosate and MCPA while the interaction effect of concentrations and cattail treatments was significant for glyphosate but not for MCPA. For each of the four cattail treatments, results indicated no significant difference in K_pl_ values under the two glyphosate concentrations, suggesting that the performances of native rhizome in absorbing glyphosate under the two concentrations were similar and that native cattail might be suitable for remediating glyphosate spills that would induce high glyphosate concentrations in water. In a previous batch equilibrium study (Hu et al. 2011), it reported that higher initial concentration of glyphosate resulted in much higher value in μg glyphosate/g sludge, which is comparable with our study when using cattail as a sorbent. Unlike the absorption of glyphosate by native rhizomes (K_pl_ > 20 L/kg), MCPA K_pl_ values remained at a relatively low level (<8 L/kg) for all cattail treatments. However, similar to the glyphosate results, regardless of whether the initial solution contained 10 μg/L or 1 mg/L MCPA, native rhizomes demonstrated significantly greater MCPA K_pl_ values than hybrid rhizomes. Thus, it is possible that in addition to glyphosate, other herbicides are less likely to be absorbed by hybrid than native rhizomes.

Our results highlight the potential of native rhizomes as a sorbent for glyphosate, giving optimal mixing conditions. Table 2 compares our sorption results to the performance of other sorbents for glyphosate and indicates that the measured K_pl_ values for native rhizomes are of the same magnitude as K values reported for other organic materials and soils.

**Table 2:** Glyphosate sorption parameters (K) determined for a range of sorbents

Sorbent	K (L/kg)	Experimental conditions					Ref.
		pH	Temperature (°C)	Time (h)	Initial glyphosate (mg/L)	Solid/solution ratio (g/mL)	
Native cattail rhizome	25.3 and 20.4	n/a	5	48	0.01 and 1	0.2/15	This study
Hybrid cattail rhizome	6.5 and 6.4	n/a	5	48	0.01 and 1	0.2/15	This study
Crop residue	6.4–256	7.4	20	n/a	0.01	1/5.8	Cassigneul et al. (2016)
Sandy loam soil	0.6–78.5	4.83–10.4	22	24	0.03–67	0.5/10	de Jonge and Wollesen de Jonge (1999)
Soil mixture	40–303	5.2–8.1	Room temperature	16	2–10	10/50	Autio et al. (2004)
Clay soil	21–87	6.3–7.5	Room temperature	70	0.1–10	1/10	Albers et al. (2009)
Clay minerals	8–138	1.8–10.0	Room temperature	1	100–200	1/25	Glass (1987)
Humic	3–17	2–7	Room temperature	70	0.2–20	0.01/1	Albers et al. (2009)
Woody biochar	88	5	Room temperature	4	20	1/1000	Mayakaduwa et al. (2016)
